# Supplementary material for: A new family of bacterial ribosome hibernation factors
Source: Nature. 2024 Feb 14;626(8001):1125–32. doi: 10.1038/s41586-024-07041-8 (PMC10901736; doi:10.1038/s41586-024-07041-8)
Supplement: Supplementary file 1 — Supplementary Table 1, Supplementary Figs 1–11 and Supplementary References. [file 41586_2024_7041_MOESM1_ESM.pdf]

nature portfolio

https://doi.org/10.1038/s41586-024-07041-8

Supplementary information

A new family of bacterial ribosome hibernation factors

In the format provided by the authors and unedited

Supplementary Information

A new family of bacterial ribosome hibernation factors

Authors

Karla Helena-Bueno[1,#], Mariia Yu. Rybak[2,#], Chinenye L. Ekemezie[1], Rudi Sullivan[3], Charlotte R. Brown[1], Charlotte Dingwall[1], Arnaud Baslé[1], Claudia Schneider[1], James P.R. Connolly[1], James N. Blaza[4,5,6], Bálint Csörgő[7], Patrick Moynihan[3], Matthieu G. Gagnon*[2,8,9,10], Chris H. Hill*[5,6,11], Sergey V. Melnikov*[1]

Affiliations

[1] Biosciences Institute, Newcastle University, Newcastle upon Tyne, UK
[2] Department of Microbiology & Immunology, University of Texas Medical Branch, USA
[3] School of Biosciences, University of Birmingham, Birmingham, UK
[4] Department of Chemistry, University of York, York, UK
[5] York Structural Biology Laboratory, University of York, York, UK
[6] York Biomedical Research Institute, University of York, York, UK
[7] Synthetic and Systems Biology Unit, Institute of Biochemistry, HUN-REN Biological Research Centre, Szeged, Hungary
[8] Department of Biochemistry & Molecular Biology, University of Texas Medical Branch, USA
[9] Sealy Center for Structural Biology & Molecular Biophysics, University of Texas Medical Branch, USA
[10] Institute for Human Infections & Immunity, University of Texas Medical Branch, USA
[11] Department of Biology, University of York, York, UK

[#] These authors contributed equally

To whom correspondence should be addressed

magagnon@utmb.edu
chris.hill@york.ac.uk
sergey.melnikov@newcastle.ac.uk

TABLE OF CONTENTS

Supplementary Tables

Supplementary Table 1 | Proteins that are structurally similar to Balon (identified by VAST)

Supplementary Figures

Supplementary Figure 1 | Validation of the cryo-EM maps for *P. urativorans* ribosomes (ice shock)

Supplementary Figure 2 | Cryo-EM maps of ribosome-bound EF-Tu isolated from ice-treated *P. urativorans*

Supplementary Figure 3 | Cryo-EM data processing workflow using CryoSPARC for *M. smegmatis* ribosomes, corresponding to **Structure 4** that comprises 70S ribosome/Msmeg1130 (as listed in **Extended Data Table 1**)

Supplementary Figure 4 | Cryo-EM data processing workflow using CryoSPARC for *M. smegmatis* ribosomes, corresponding to **Structure 5** that comprises 70S ribosome/Rv2629 (as listed in **Extended Data Table 1**)

Supplementary Figure 5 | Comparison of the molecular features of Balon, Msmeg1130, and Rv2629

Supplementary Figure 6 | Comparison of growth curves of wild-type and Balon-deficient *P. arcticus*

Supplementary Figure 7 | Analysis of total RNA, including rRNA, from the wild-type and Balon-deficient *P. arcticus* strains under normal growth conditions and after long-term stationary phase

Supplementary Figure 8 | Balon binding site has multiple conformations during the ribosomal active cycle

Supplementary Figure 9 | Recombinant *M. smegmatis* EF-Tu expressed in *E. coli* co-purifies with GDP

Supplementary Figure 10 | Size exclusion chromatography to measure the association between EF-Tu and the Balon homolog Msmeg1130 (control experiments)

Supplementary Figure 11 | Co-sedimentation assays to assess the association of *M. smegmatis* ribosomes with Rv2629, Msmeg1130 and EF-Tu in the presence of GDP, GDPCP or GTP

Supplementary Table

Supplementary Table 1 | Proteins that are structurally similar to Balon (identified by VAST). The table illustrates that, of all structurally characterized proteins, Balon is the most structurally similar to proteins Pelota and aeRF1 from the archaeo-eukaryotic branch of life.

PDB ID	Balon segment	Aligned residues	P-value	RMSD (Å)	Sequence identity (%)	Protein	Organism
3OBY	All structure	193	10e-6.2	4.0	15.5	Pelota	*Archaeoglobus fulgidus*
3WXM	Middle domain	117	0.0004	3.7	12.8	Pelota	*Aeropyrum pernix*
3IR9	All structure	104	0.0023	2.6	12.5	aeRF1	*Methanosarcina mazei*
2QI2	Middle domain	101	10e-5.4	3.0	12.9	Pelota	*Thermoplasma acidophilum*

Supplementary Figures

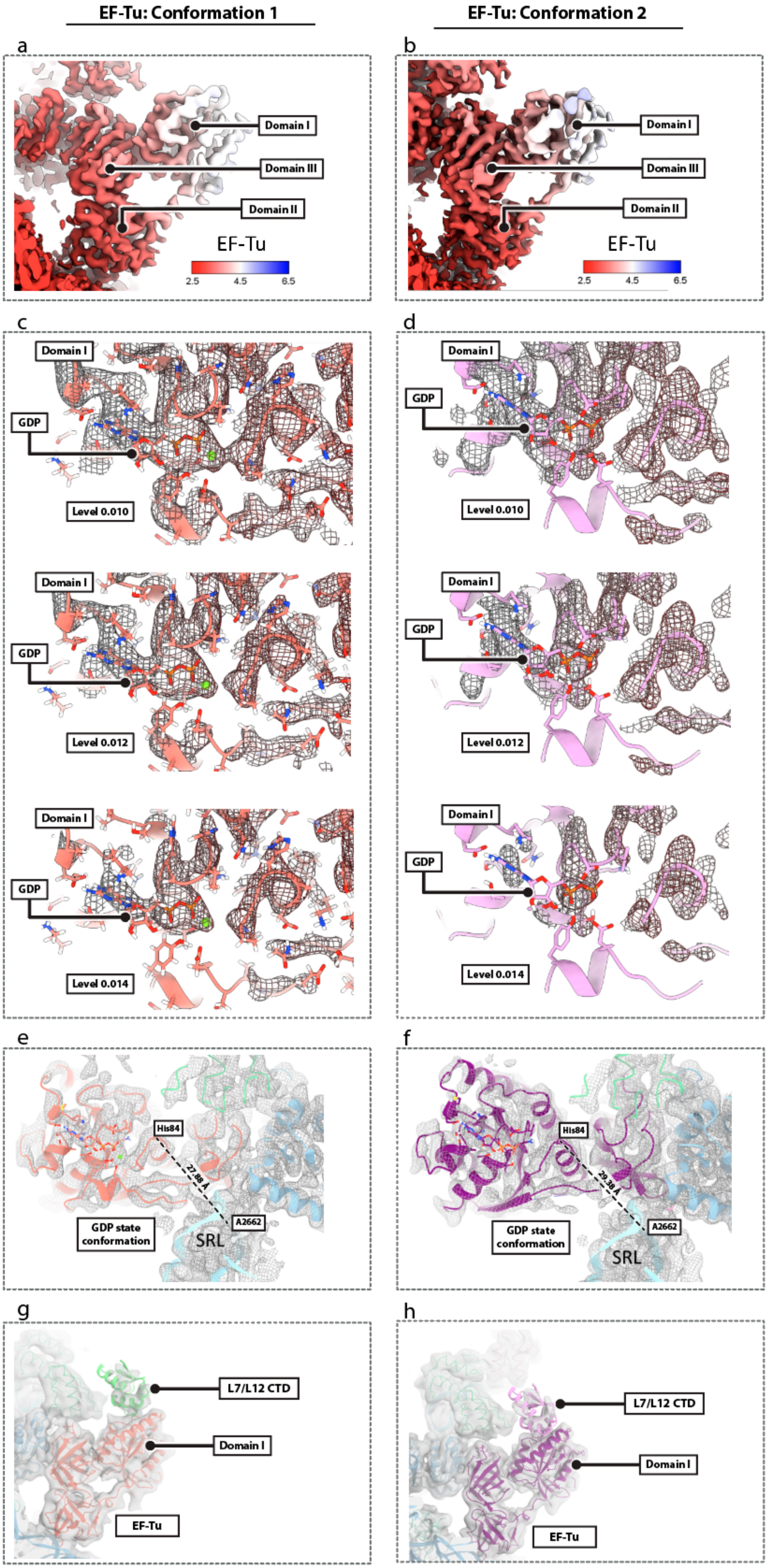

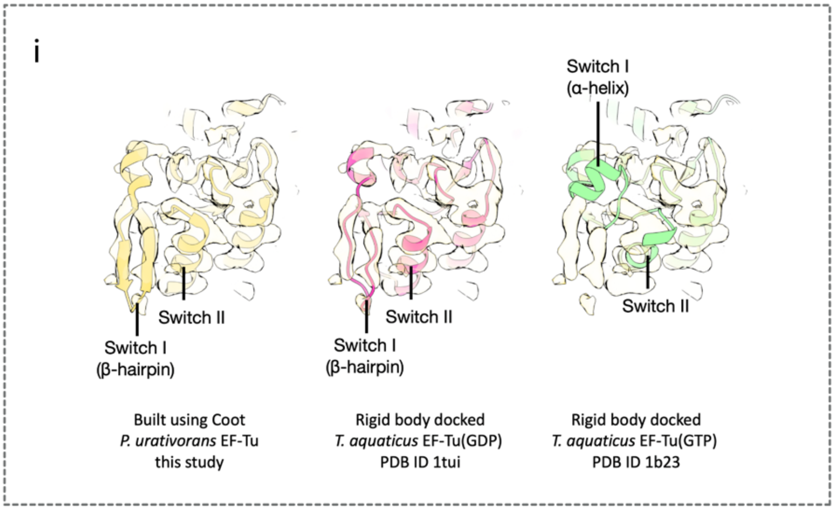

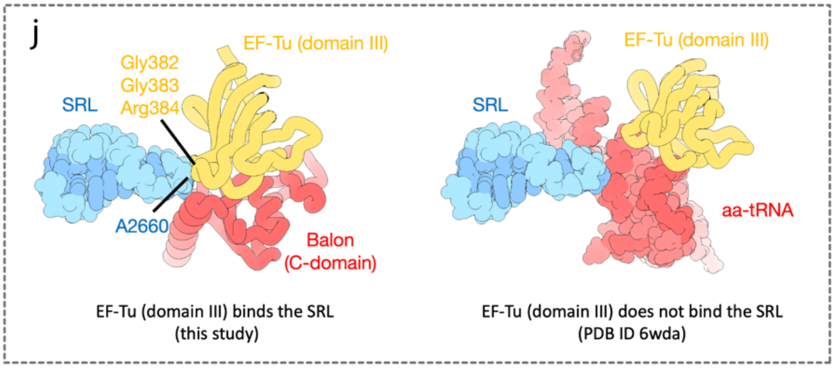

Supplementary Figure 1 | Cryo-EM maps of ribosome-bound EF-Tu isolated from ice-treated *P. urativorans*. The ribosome-bound EF-Tu conformations 1 and 2, and the corresponding panels are arranged in pairs, with conformation 1 on the left and conformation 2 on the right. (**a, b**) Cryo-EM maps coloured by local resolution, showing the overall shape of the ribosome-bound EF-Tu in conformations 1 and 2. (**c, d**) Zoomed-in views of domain I of EF-Tu, showing a GDP/Mg^{2+} model in the nucleotide binding pocket in conformation 1 and GDP in conformation 2. (**e, f**) Cryo-EM maps showing the overall shape of the nucleotide-binding domain I of EF-Tu. (**g, h**) Zoomed-in views of ribosome-bound EF-Tu in conformations 1 and 2, showing the apparent association of domain I of EF-Tu with the C-terminal domain of bL12 in the L7/L12-stalk. Cryo-EM maps were filtered to 6 Å for clarity. (**i**) Cryo-EM density corresponding to domain I of EF-Tu in the better-resolved conformation I of *P. urativorans* EF-Tu associated with Balon-bound ribosomes. The panel compares the structure of EF-Tu switches determined in this study with the structures of switches in the open GDP-bound conformation of EF-Tu (PDB ID 1tui)[1] and the closed GTP-bound conformation of EF-Tu (PDB ID 1b23)[2]. The correspondence between the density and the structures illustrate that, upon binding to Balon and the ribosome, EF-Tu adopts the GDP-type conformation of switches I and II rather than the GTP-type conformation. (**j**) Zoom-in views illustrate that upon EF-Tu binding to Balon and the ribosome, domain III of EF-Tu binds to the sarcin-ricin loop of the ribosome (SRL). However, in the previously observed structures of EF-Tu-dependent delivery of aminoacyl-tRNA to the ribosomal A site, domain III of EF-Tu remains detached from the sarcin-ricin loop. For simplicity, only one structure of the EF-Tu/aminoacyl-tRNA complex is shown, however essentially the same position of domain III (rmsd < 2 Å) has been observed for all intermediate states of EF-Tu in structures with PDB IDs 6wd2-6wd9 and 6wda[3].

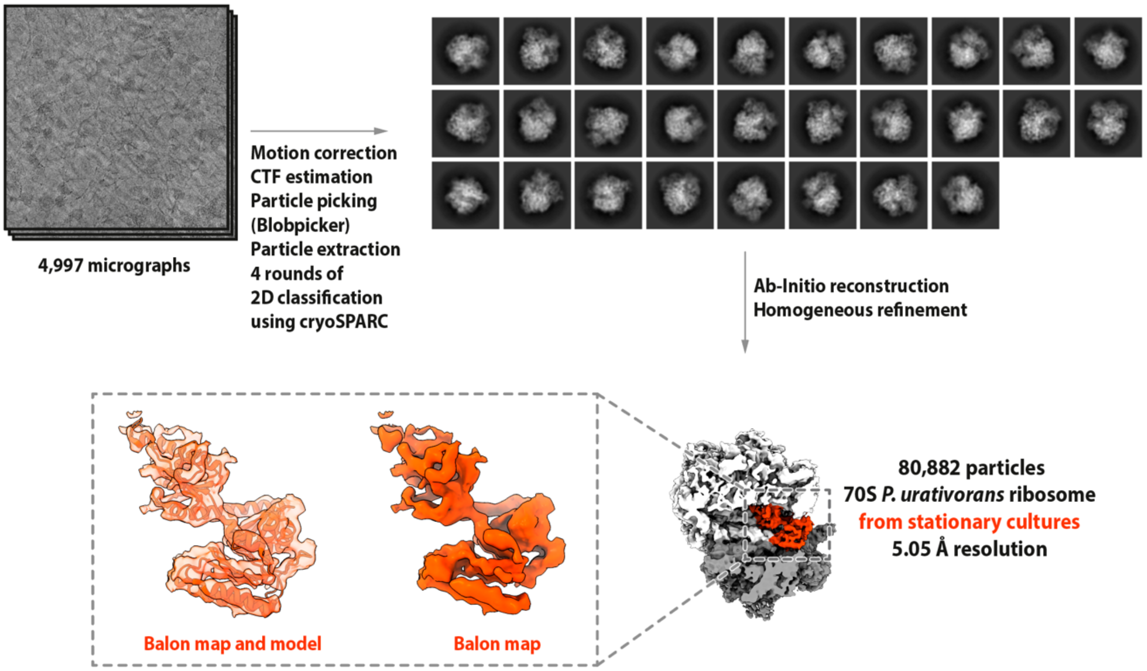

Supplementary Figure 2 | Cryo-EM data processing workflow for the analysis of *P. urativorans* ribosomes isolated from stationary bacterial cultures (Dataset 2 – stationary phase). The pipeline shows a representative micrograph at 150,000x with 2D classes, 3D reconstructions and major steps of data processing using CryoSPARC. The figure shows that *P. urativorans* ribosomes bind with Balon not only during ice treatment but also during stationary phase, suggesting that Balon serves as a general stress response hibernation factor instead of its activity being limited to one specific stress condition (i.e. ice treatment).

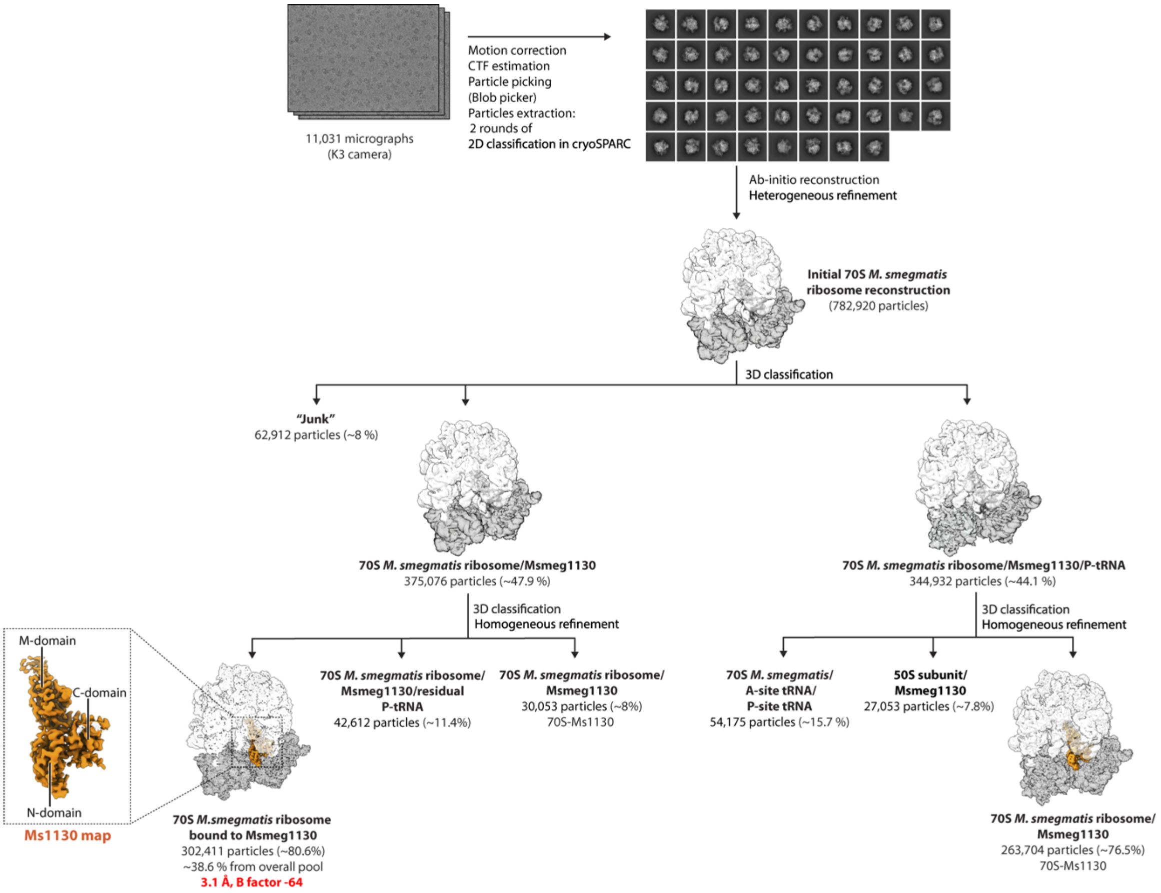

Supplementary Figure 3 | Cryo-EM data processing workflow using CryoSPARC for *M. smegmatis* ribosomes, corresponding to Structure 4 that comprises 70S ribosome/Msmeg1130 (as listed in Extended Data Table 1).

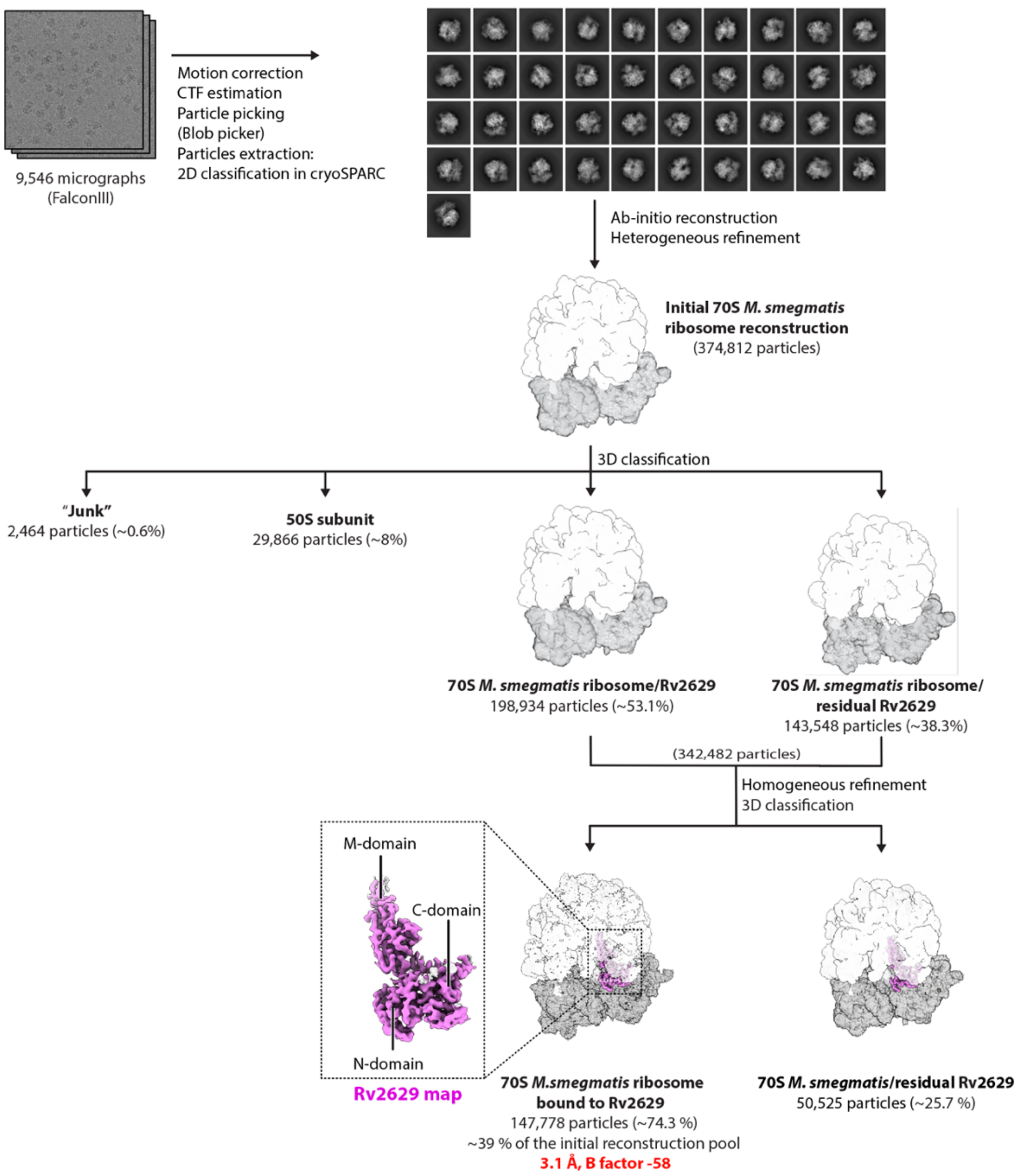

Supplementary Figure 4 | Cryo-EM data processing workflow using CryoSPARC for *M. smegmatis* ribosomes, corresponding to Structure 5 that comprises 70S ribosome/Rv2629 (as listed in Extended Data Table 1).

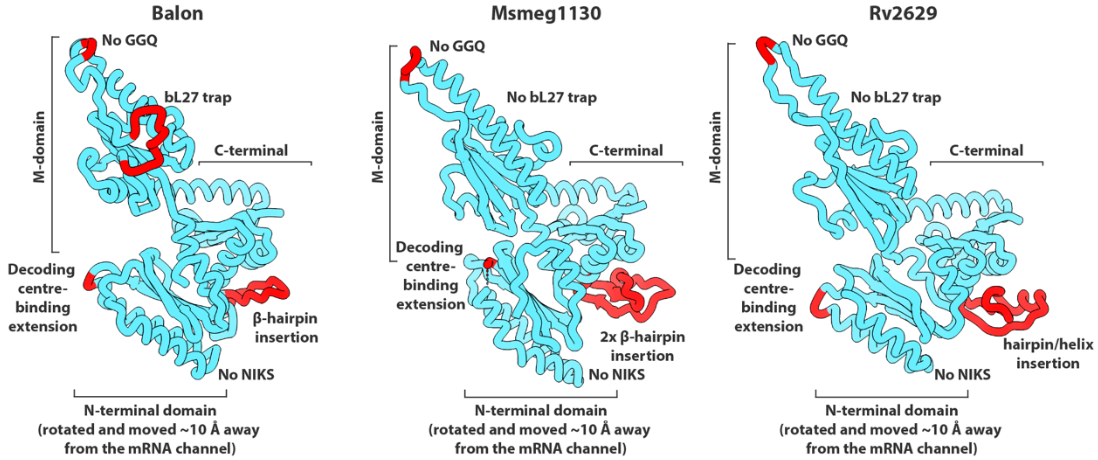

Supplementary Figure 5 | Comparison of the molecular features of Balon, Msmeg1130, and Rv2629. Structures of Balon from *P. urativorans*, Msmeg1130 from *M. smegmatis* and Rv2629 from *M. tuberculosis* are shown in their aligned views and are derived from the structures of these factors bound to 70S ribosomes determined in this study. Balon, Msmeg1130, and Rv2629 bear the same structural characteristics that include: (i) the lack of the NIKS motif in their mRNA-binding N-terminal domain and the presence of the extended loop on the opposite side of the N-terminal globule that allow these factors to directly bind the decoding centre of the ribosome (i.e. HP motif in Balon); (ii) the lack of the GGQ motif in their peptidyl-transferase-binding middle domain, (iii) and the characteristic insertion in their EF-Tu-binding C-terminal domain (β-hairpin insertion in Balon referred to as “β-loop” in this study, and similar but larger insertions in Balon homologs from *Mycobacteria* sp.).

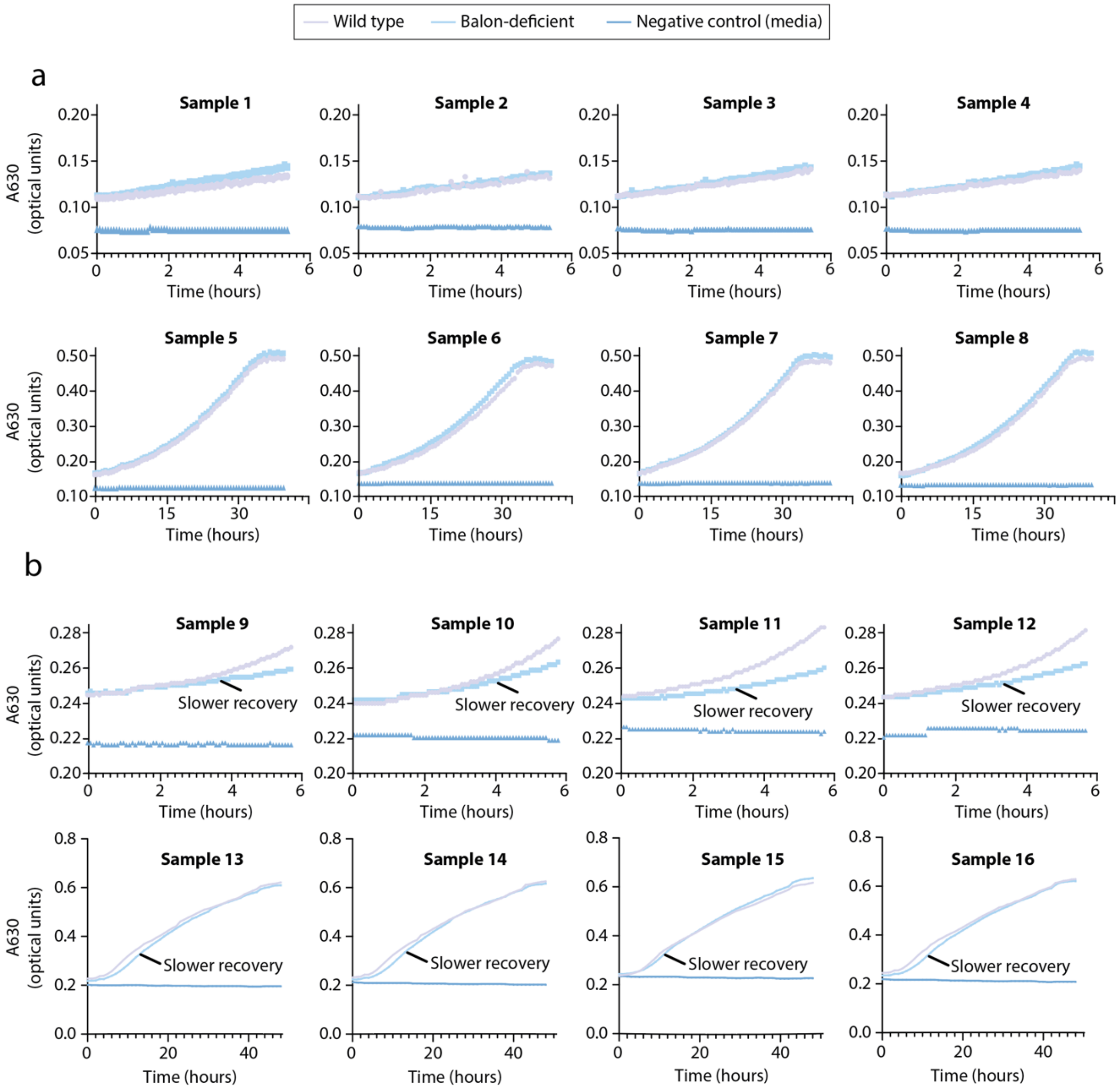

Supplementary Figure 6 | Comparison of growth curves of wild-type and Balon-deficient *P. arcticus*. (**a**) Plots compare the normal growth of two strains of *P. arcticus* (wild-type versus Balon-deficient). For each experiment, 0.1 mL aliquots of four independent and actively growing *P. arcticus* cultures (A_{630} ~0.8) were diluted in 1 mL of Marine Broth media. Then, samples 1-4 were observed during the first 6 hours of growth and illustrate that the Balon-deficient *P. arcticus* strain appears to grow slightly faster compared to the wild-type strain. Samples 5-8 were observed for 2 days and illustrate that the Balon-deficient *P. arcticus* strain appears to produce a higher A_{630} upon reaching the stationary phase compared to the wild-type strain. (**b**) Plots compare the recovery of two strains of *P. arcticus* (wild-type versus Balon-deficient) after three months of continuous stationary phase. For each experiment, 0.1 mL aliquots of the stationary-phase cultures (A_{630} ~1.5) were diluted in 1 mL of Marine Broth 2216 media. Samples 9-12 were observed during the first 6 hours of growth recovery and illustrate that the wild-type strain grows faster compared to the Balon-deficient strain during the first few hours of recovery from stationary phase. Samples 13-16 were observed for 48 hours and illustrate that the Balon-deficient cultures eventually “catch up” with the wild-type cultures as cells approach the stationary phase. All the experiments in (**a**, **b**) were conducted while incubating and shaking the samples in the BioTEK 800TS plate reader at room temperature using 24-well plates sealed with Breathe-Easy® sealing membranes to allow for sample oxygenation. The room temperature varied between 20 and 26 °C for the following sets of samples, which were tested on four separate days: (1-4), (5-8), (9-12), and (13-16).

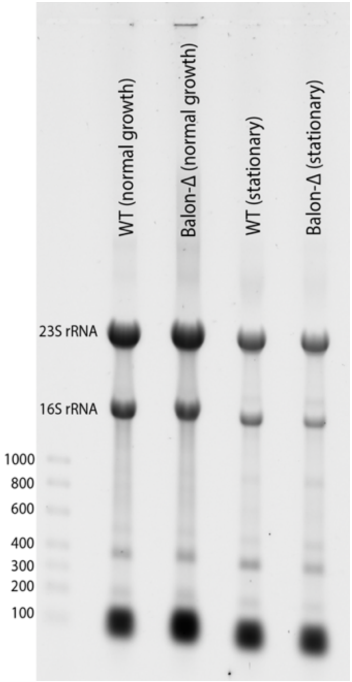

Supplementary Figure 7 | Analysis of total RNA, including rRNA, from the wild-type and Balon-deficient *P. arcticus* strains under normal growth conditions and after long-term stationary phase. The gel image illustrates the relative abundance of 23S and 16S rRNA isolated from equal masses (0.1 g) of four samples of *P. arcticus* cells: the wild-type and Balon-deficient strains under normal growth conditions and after three months of continuous stationary phase. The numbers next to the DNA ladder indicate the lengths of the DNA fragments in base pairs.

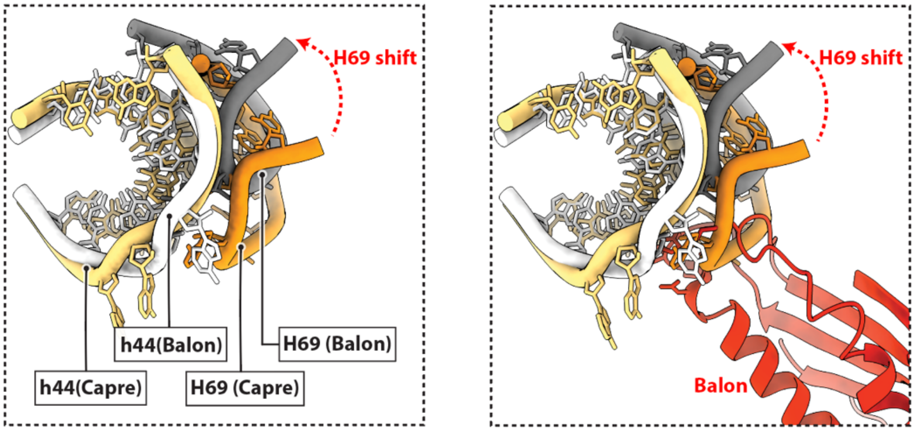

Supplementary Figure 8 | Balon binding site has multiple conformations during the ribosomal active cycle. To recognize the decoding centre of the ribosome, Balon binds at the interface of two rRNA helices: 16S helix h44 and 23S helix H69. These two helices are known to adapt various conformations relative to each other. The figure shows superposition of two structures in which ribosomes adopt either a non-rotated conformation (as observed in this study) or a rotated conformation (e.g. the one shown in this figure that represents the ribosome structure with the antibiotic capreomycin, PDB ID 4v7m)[4]. In the rotated state of the ribosome, H69 is shifted relative to h44, and H69 would clash with the N-terminal domain of Balon, indicating that Balon cannot use the same recognition strategy to bind this conformation of the decoding centre.

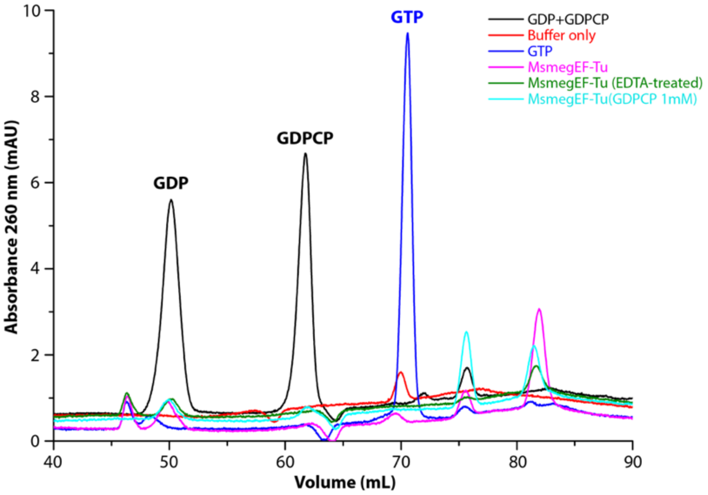

Supplementary Figure 9 | Recombinant *M. smegmatis* EF-Tu expressed in *E. coli* co-purifies with GDP. Reverse-phase HPLC analysis illustrates the retention time of individual nucleotides GDP and GDPCP (black trace) and GTP (blue trace). The magenta trace (labelled as MsmegEF-Tu) corresponds to EF-Tu from *M. smegmatis* that was recombinantly expressed and isolated from *E. coli*, illustrating that this EF-Tu sample is bound to residual amounts of GDP. The green trace (labelled as MsmegEF-Tu (EDTA-treated)) corresponds to EDTA-treated EF-Tu, representing our attempt to remove residual amounts of GDP with EDTA. This green trace shows that EF-Tu remains bound to residual amounts of GDP despite the EDTA treatment. The cyan trace (labelled as MsmegEF-Tu + 1 mM GDPCP) corresponds to EF-Tu incubated with 1 mM GDPCP for 5 minutes at 37 °C, followed by purification on a PD MiniTrap G-25 column and treatment with perchloric acid (these experimental conditions were used to prepare the complex of *M. smegmatis* ribosomes with Msmeg1130 and EF-Tu in the presence of GDPCP). This cyan trace indicates that EF-Tu remains bound to residual amounts of GDP even after the pre-incubation with GDPCP. All the experiments were conducted using a PROTO 300 C4 column and a 0–30% gradient of acetonitrile in buffer containing 100 mM KH_2PO_4, pH 6.5, and 10 mM tetrabutylammonium hydrogensulfate.

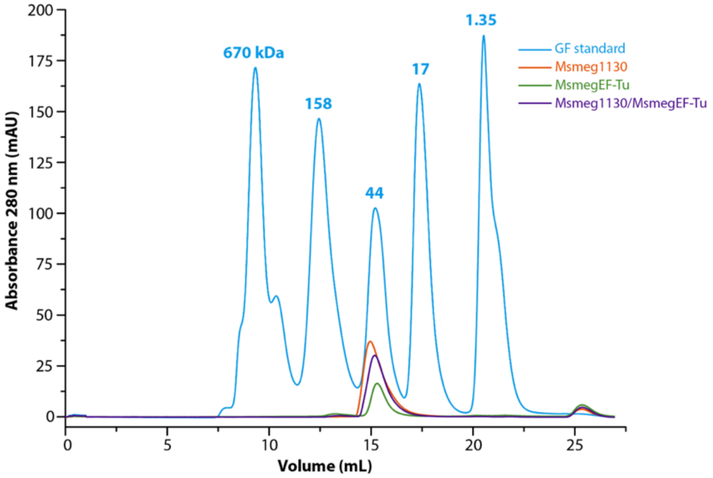

Supplementary Figure 10 | Size exclusion chromatography to measure the association between EF-Tu and the Balon homolog Msmeg1130 (control experiments). The plot shows the control experiments in which the retention volume values were determined for individual proteins MsmegEF-Tu and Msmeg1130 and their mixture in the absence of any nucleotides (labelled as Msmeg1130/MsmegEF-Tu). The blue trace shows the retention volumes for standard protein molecules, with the peaks labelled by their molecular weight in kDa. Note that MsmegEF-Tu and Msmeg1130 have very similar molecular weights (41 and 43 kDa, respectively) and similar retention volumes, and mixing these proteins together does not decrease their retention volume, indicating the lack of stable interaction between the two proteins.

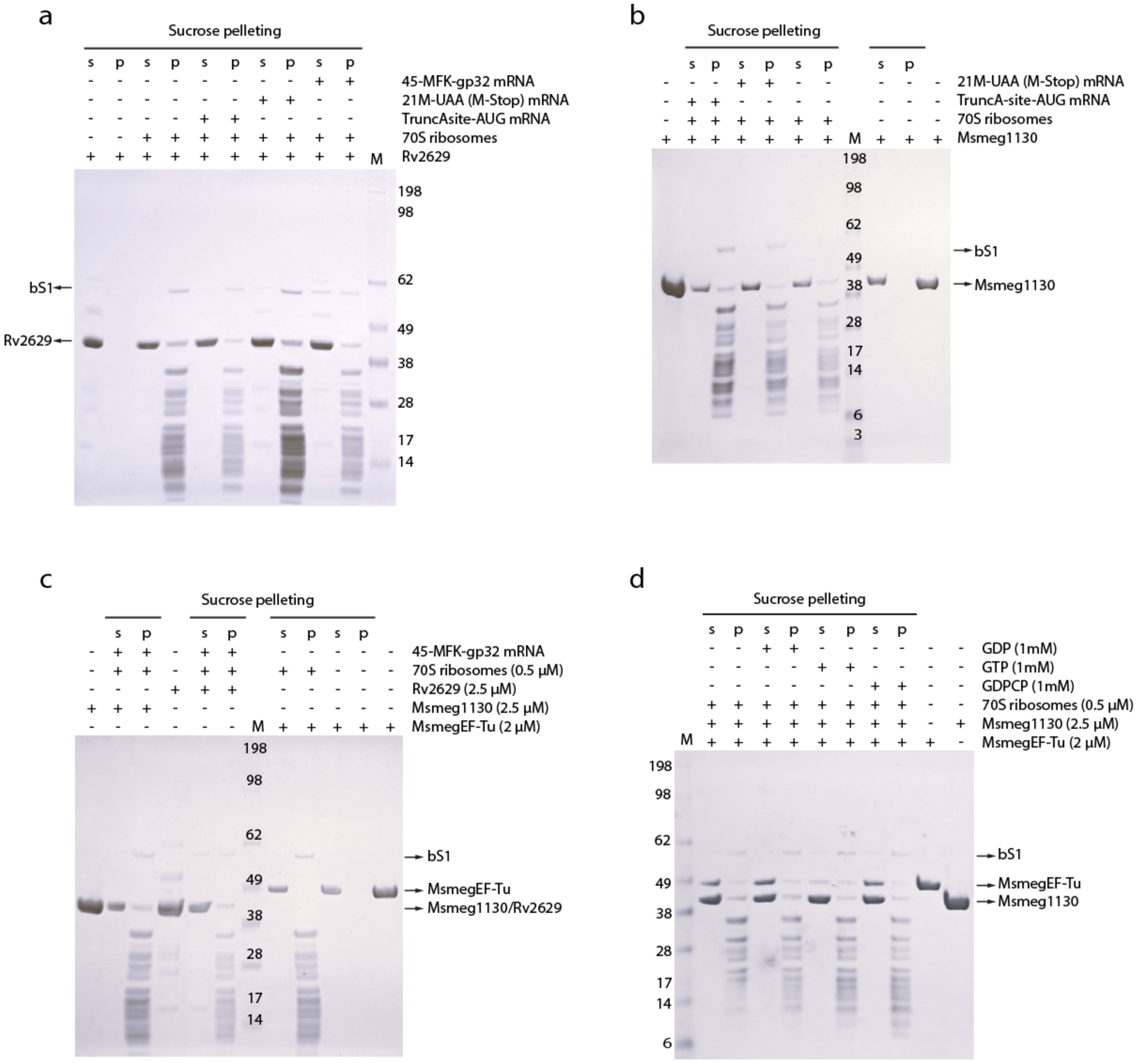

Supplementary Figure 11 | Co-sedimentation assays to assess the association of *M. smegmatis* ribosomes with Rv2629, Msmeg1130 and EF-Tu in the presence of GDP, GDPCP or GTP. In all panels, EF-Tu is labelled as MsmegEF-Tu to indicate the use of EF-Tu from *M. smegmatis* that was recombinantly expressed and purified from *E. coli*. (**a**) Evaluation of the binding of *M. tuberculosis* Rv2629 and (**b**) *M. smegmatis* 1130 to *M. smegmatis* 70S ribosomes in the presence of various mRNAs, including truncated A-site AUG mRNA, 21-Met-UAA (M-Stop), and long 45-MPK-gp32-mRNA, showing that Rv2629 and Msmeg1130 bind in an mRNA-independent manner. (**c, d**) Investigation of the binding of Msmeg1130 and EF-Tu to 70S ribosomes in the presence or absence of each of the three nucleotides, including GDP, GDPCP (guanosine 5′-β,γ-methylenetriphosphate) and GTP. *M. smegmatis* mc²155 70S ribosomes (final concentration 0.5 μM) were programmed (**a, b**) with mRNA (1.25 μM) by incubation at 37 °C for 5 minutes in 1X buffer B (20 mM HEPES-KOH pH 7.5, 60 mM KCl, 10 mM $MgCl_2$, 1 mM DTT). Subsequently, Msmeg1130 or Rv2629 (2.5 μM) was added and incubated for 15 minutes at room temperature. For complexes with Msmeg1130 and EF-Tu (**c, d**), 2 μM EF-Tu was pre-incubated with 1 mM GDP or GDPCP or GTP for 5 minutes at 37 °C, then added to 70S ribosomes with 2.5 μM Msmeg1130. The mixture was centrifuged through a cold sucrose cushion buffer (20 mM HEPES-KOH pH 7.5, 60 mM KCl, 10 mM $MgCl_2$, 1 mM DTT, 30% sucrose), in the TLS55 rotor (Beckman Coulter) for 1 hour at 214,000 x *g* at 15 °C. The top 50 μl sample solution and the bottom 20 μl were precipitated with 100% trichloroacetic acid (TCA), pelleted by centrifugation, washed with ice-cold acetone, and dissolved in 1X SDS-protein loading buffer. The results were analysed via 4-12% SDS-PAGE gel (SurePage™, GenScript).

Supplementary references

1 Polekhina, G., Thirup, S., Kjeldgaard, M., Nissen, P., Lippmann, C. & Nyborg, J. Helix unwinding in the effector region of elongation factor EF-Tu–GDP. *Structure* **4**, 1141-1151, doi.org/10.1016/S0969-2126(96)00122-0 (1996).

2 Nissen, P., Kjeldgaard, M., Thirup, S. & Nyborg, J. The crystal structure of Cys-tRNACys–EF-Tu–GDPNP reveals general and specific features in the ternary complex and in tRNA. *Structure* **7**, 143-156, doi.org/10.1016/S0969-2126(99)80021-5 (1999).

3 Loveland, A. B., Demo, G. & Korostelev, A. A. Cryo-EM of elongating ribosome with EF-Tu*GTP elucidates tRNA proofreading. *Nature* **584**, 640-645, doi:10.1038/s41586-020-2447-x (2020).

4 Stanley, R. E., Blaha, G., Grodzicki, R. L., Strickler, M. D. & Steitz, T. A. The structures of the anti-tuberculosis antibiotics viomycin and capreomycin bound to the 70S ribosome. *Nature structural & molecular biology* **17**, 289-293, doi.org/10.1038/nsmb.1755 (2010).
