# Supplementary material for: A new family of bacterial ribosome hibernation factors
Source: Nature. 2024 Feb 14;626(8001):1125–32. doi: 10.1038/s41586-024-07041-8 (PMC10901736; doi:10.1038/s41586-024-07041-8)
Supplement: Supplementary file 2 — Reporting Summary [file 41586_2024_7041_MOESM2_ESM.pdf]

Corresponding author(s): Sergey V. Melnikov
Chris H. Hill
Matthieu G. Gagnon

Last updated by author(s): Nov 21, 2021

Reporting Summary

Nature Portfolio wishes to improve the reproducibility of the work that we publish. This form provides structure for consistency and transparency in reporting. For further information on Nature Portfolio policies, see our Editorial Policies and the Editorial Policy Checklist.

Statistics

For all statistical analyses, confirm that the following items are present in the figure legend, table legend, main text, or Methods section.

n/a	Confirmed	
☐	☒	The exact sample size (*n*) for each experimental group/condition, given as a discrete number and unit of measurement
☐	☒	A statement on whether measurements were taken from distinct samples or whether the same sample was measured repeatedly
☒	☐	The statistical test(s) used AND whether they are one- or two-sided<br>*Only common tests should be described solely by name; describe more complex techniques in the Methods section.*
☒	☐	A description of all covariates tested
☒	☐	A description of any assumptions or corrections, such as tests of normality and adjustment for multiple comparisons
☒	☐	A full description of the statistical parameters including central tendency (e.g. means) or other basic estimates (e.g. regression coefficient) AND variation (e.g. standard deviation) or associated estimates of uncertainty (e.g. confidence intervals)
☒	☐	For null hypothesis testing, the test statistic (e.g. *F*, *t*, *r*) with confidence intervals, effect sizes, degrees of freedom and *P* value noted<br>*Give P values as exact values whenever suitable.*
☒	☐	For Bayesian analysis, information on the choice of priors and Markov chain Monte Carlo settings
☒	☐	For hierarchical and complex designs, identification of the appropriate level for tests and full reporting of outcomes
☒	☐	Estimates of effect sizes (e.g. Cohen's *d*, Pearson's *r*), indicating how they were calculated

Our web collection on statistics for biologists contains articles on many of the points above.

Software and code

Policy information about availability of computer code

Data collection	The cryo-EM data were collected using the Smart EPU Software for single particle analysis by Thermofisher Scientific. The bacterial growth curves were recordered using the Agilent BioTek Gen 5 software package.
Data analysis	The models were created using Coot v0.8.9.2<br>The models were analyzed and visualized using ChimeraX v1.4 and Chimera v1.14<br>The cryo-EM snapshots were processed using Relion v3.1 and CryoSPARC v4.3.0<br>The models were refined using Phenix v1.20.1-4487

For manuscripts utilizing custom algorithms or software that are central to the research but not yet described in published literature, software must be made available to editors and reviewers. We strongly encourage code deposition in a community repository (e.g. GitHub). See the Nature Portfolio guidelines for submitting code & software for further information.

Data

Policy information about availability of data

All manuscripts must include a data availability statement. This statement should provide the following information, where applicable:

- Accession codes, unique identifiers, or web links for publicly available datasets
- A description of any restrictions on data availability
- For clinical datasets or third party data, please ensure that the statement adheres to our policy

DATA AVAILABILITY
The Supplementary Data 1-9 presented in this manuscript are freely accessible and available through the public repository of data, FigShare, using the following link: https://figshare.com/s/374a95769c5f7e9cdc04. The PDB accession codes for Structures 1-6, along with the associated cryo-EM maps will be made available before the publication of this manuscript via the Protein Data Bank. Currently, these structural data are shared through a freely accessible link on Google Drive.

Research involving human participants, their data, or biological material

Policy information about studies with human participants or human data. See also policy information about sex, gender (identity/presentation), and sexual orientation and race, ethnicity and racism.

Reporting on sex and gender	We did not study any human populations in our study.
Reporting on race, ethnicity, or other socially relevant groupings	We did not study any human populations in our study.
Population characteristics	We did not study any human populations in our study.
Recruitment	We did not study any human populations in our study.
Ethics oversight	We did not study any human populations in our study.

Note that full information on the approval of the study protocol must also be provided in the manuscript.

Field-specific reporting

Please select the one below that is the best fit for your research. If you are not sure, read the appropriate sections before making your selection.

☒ Life sciences ☐ Behavioural & social sciences ☐ Ecological, evolutionary & environmental sciences

For a reference copy of the document with all sections, see nature.com/documents/nr-reporting-summary-flat.pdf

Life sciences study design

All studies must disclose on these points even when the disclosure is negative.

Sample size	Each cryo-EM map presented in this study was calculated by using at least 10,287 individual ribosome snapshots to reconstruct the three-dimensional volume of the ribosome.
Data exclusions	Some ribosome images were excluded from the analysis based on the signal corresponding to the ribosomal ligands in the masked regions as described in detail in the Materials and Methods and Supplementary Figure 2.
Replication	To measure the impact of deletions of the Balon-coding gene on P. arcticus growth, each measurement was made eight times using different cell samples for each measurement.
Randomization	N/A
Blinding	N/A

Reporting for specific materials, systems and methods

We require information from authors about some types of materials, experimental systems and methods used in many studies. Here, indicate whether each material, system or method listed is relevant to your study. If you are not sure if a list item applies to your research, read the appropriate section before selecting a response.

Materials & experimental systems

n/a	Involved in the study
☒	☐ Antibodies
☒	☐ Eukaryotic cell lines
☒	☐ Palaeontology and archaeology
☒	☐ Animals and other organisms
☒	☐ Clinical data
☒	☐ Dual use research of concern
☒	☐ Plants

Methods

n/a	Involved in the study
☒	☐ ChIP-seq
☒	☐ Flow cytometry
☒	☐ MRI-based neuroimaging

Plants

Seed stocks

We did not study plants in our study.

Novel plant genotypes

We did not study plants in our study.

Authentication

We did not study plants in our study.
